# Supplementary material for: Mechanism of fertilization-induced auxin synthesis in the endosperm for seed and fruit development
Source: Nat Commun. 2022 Jul 9;13:3985. doi: 10.1038/s41467-022-31656-y (PMC9271072; doi:10.1038/s41467-022-31656-y)
Supplement: Supplementary file 1 — Supplementary Information [file 41467_2022_31656_MOESM1_ESM.pdf]

Supplementary Information

Title: Mechanism of fertilization-induced auxin synthesis in the endosperm for seed and fruit development

Lei Guo[1], Xi Luo[1], Muzi Li[1], Dirk Joldersma[1], Madison Plunkert[1], and Zhongchi Liu[1,*]

[1]Department of Cell Biology and Molecular Genetics, University of Maryland, College Park, MD 20742, USA

*Corresponding author:
Zhongchi Liu
Email: zliu@umd.edu

Supplementary Figures: 11

Supplementary Table: 1

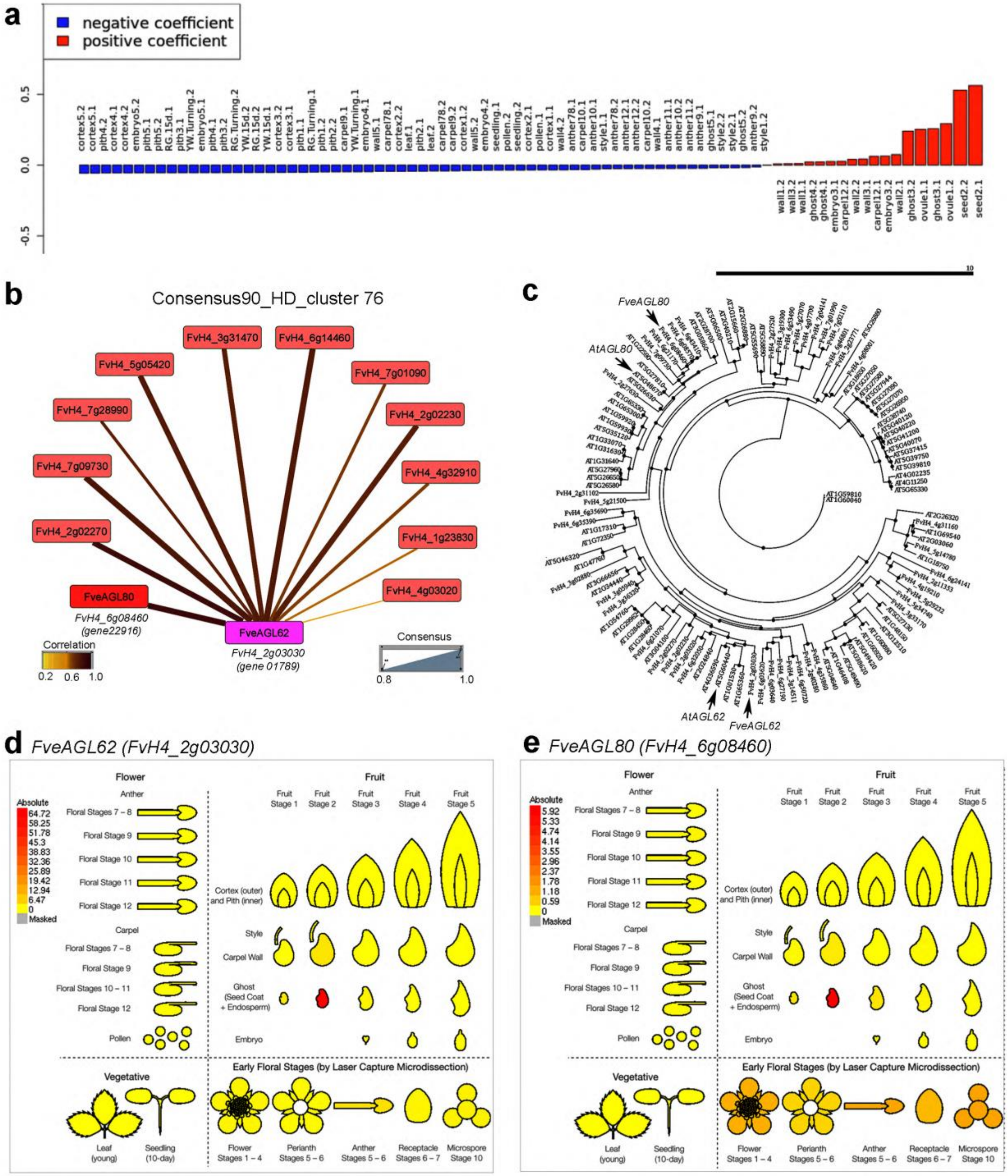

Supplementary Figure 1. Bioinformatic analysis of two Class I MADS-box genes *FveAGL62* and *FveAGL80* in *F.vesca.* **a** Cluster eigengene value across different tissues (hand-dissected RNA-seq samples) of the consensus co-expression cluster 76[1]. The cluster eigengene is most highly expressed in the stage 2 seed. **b** Correlation of *FveAGL62* and *FveAGL80* with other 11 co-expressed transcription factor genes in cluster 76. Edge color and width indicate correlation and consensus coefficient, respectively. **c** Phylogenetic analysis of type I MADS genes from

Arabidopsis and *F. vesca*. Arabidopsis *AtAGL62* and *AtAGL80*, and strawberry *FveAGL62* and *FveAGL80* are indicated with arrows. **d**-**e** eFP gene expression heatmap of *FveAGL62* (**d**) and *FveAGL80* (**e**). Note the transient activation of *FveAGL62* and *FveAGL80* at stage 2 seed. The expression value in the scale bar is TPM (Transcripts Per Million). The images in d-e are screenshots of the strawberry eFP browser published by Hawkins et al[2] with a title "An eFP browser for visualizing strawberry fruit and flower transcriptomes" under the terms of Creative Commons CC BY license.

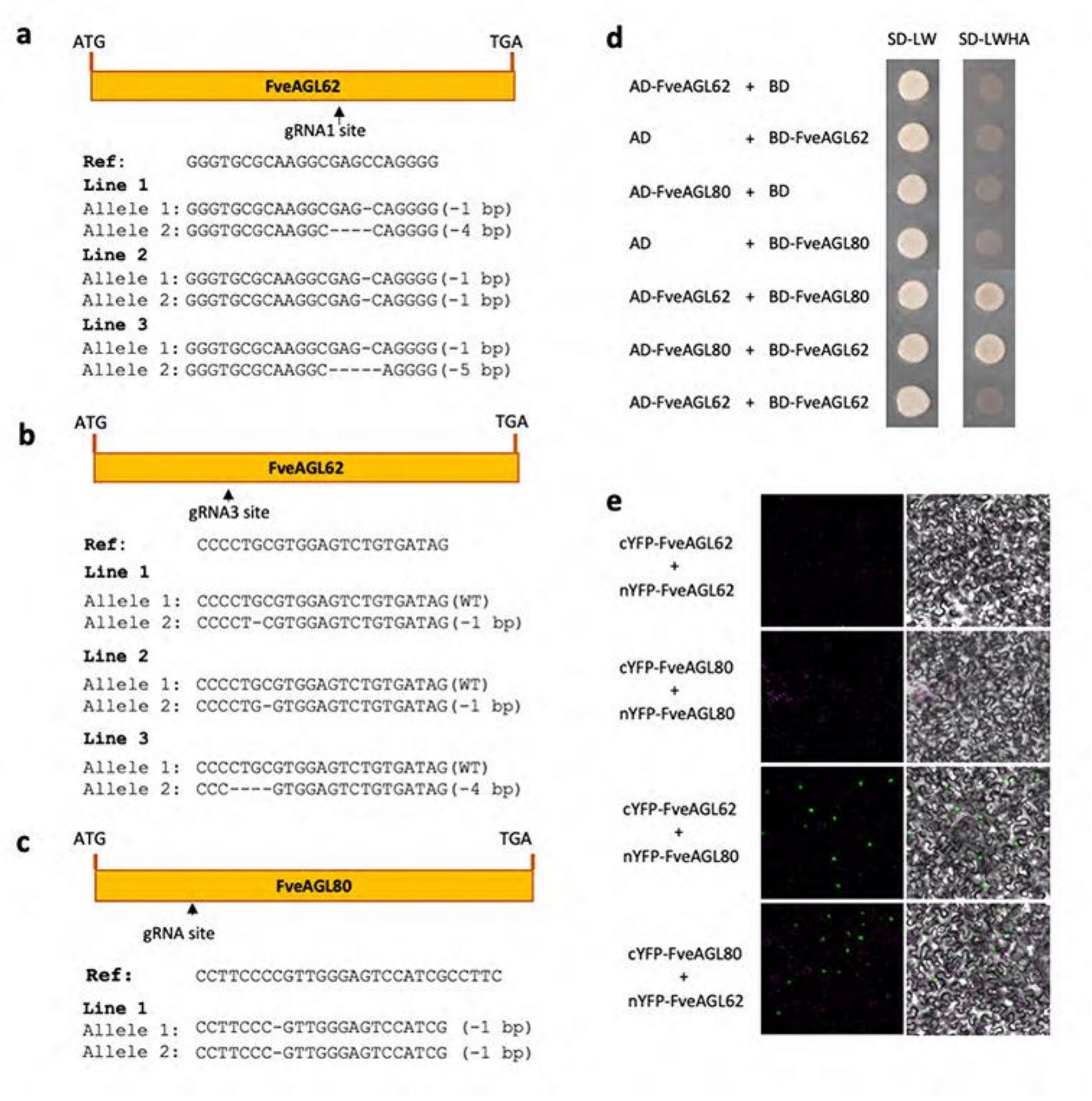

Supplementary Figure 2. Analyses of CRISPR-knockout mutants and protein-protein interactions of *FveAGL62* and *FveAGL80*. a Analyses of Cas9-sgRNA1-induced mutations in *FveAGL62*; three biallelic or homozygous mutant lines are shown. **b** Analysis of Cas9-sgRNA3-induced mutations in a different region of *FveAGL62* via independent transformation. Three heterozygous lines are shown. **c** A single homozygous mutant line of *FveAGL80* is shown. **d** Results of the Y2H assay showing interaction between FveAGL62 and FveAGL80 and a lack of homodimerization of FveAGL62. **e** Results of a BiFC assay showing the interaction between FveAGL62 and FveAGL80. Neither FveAGL62 nor FveAGL80 homodimerizes.

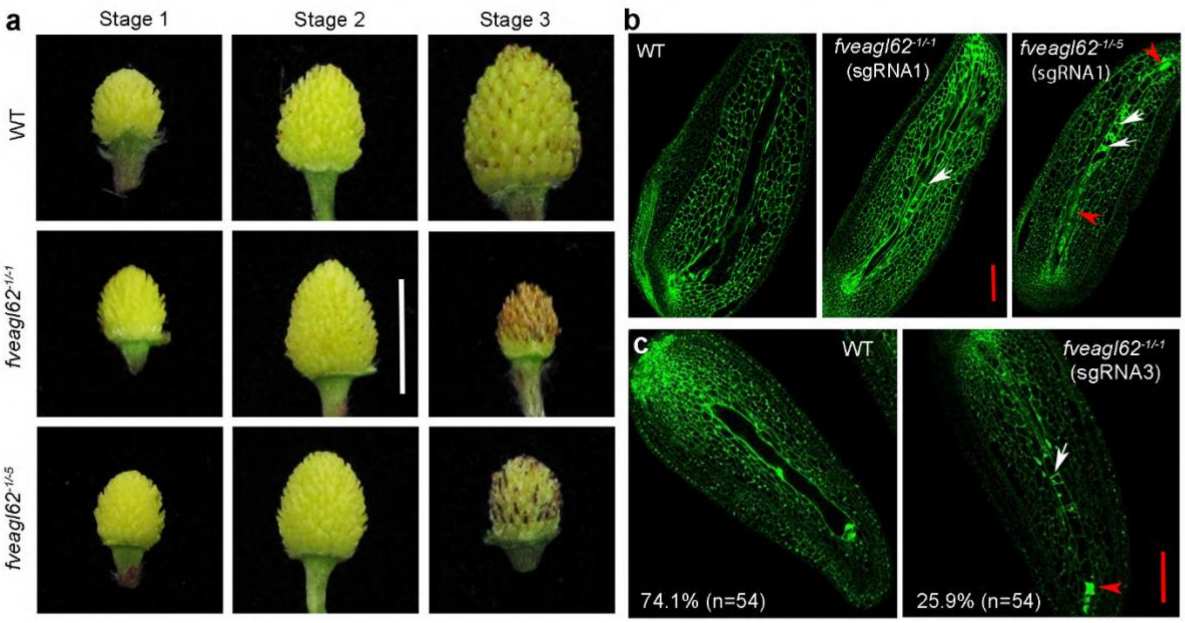

Supplementary Figure 3. Characterization of additional CRISPR *fveagl62* mutant lines. a Comparison of receptacle fruit development at the three earliest stages of fruit development. *fveagl62*$^{-1/-1}$ and *fveagl62*$^{-1/-5}$ are independent mutant lines generated by the Cas9-sgRNA1 (see Supplementary Fig. 2a). **b** Confocal laser scanning microscopy images of WT and *fveagl62* mutant seeds at stage 2. Precocious cellularization of *fveagl62* endosperms (arrows) are observed in both *fveagl62*$^{-1/-1}$ and *fveagl62*$^{-1/-5}$ stage 2 seeds. **c** Confocal image of stage 2 seeds from an *fveagl62*$^{-1/+}$ heterozygous parent. The mutation is generated with Cas9-sgRNA3 targeting a different region of *FveAGL62*. Both wild type (74.1%) and mutant endosperm (25.9%) phenotype are observed. Scale bars, 5 mm (**a**), 100 μm (**b, c**).

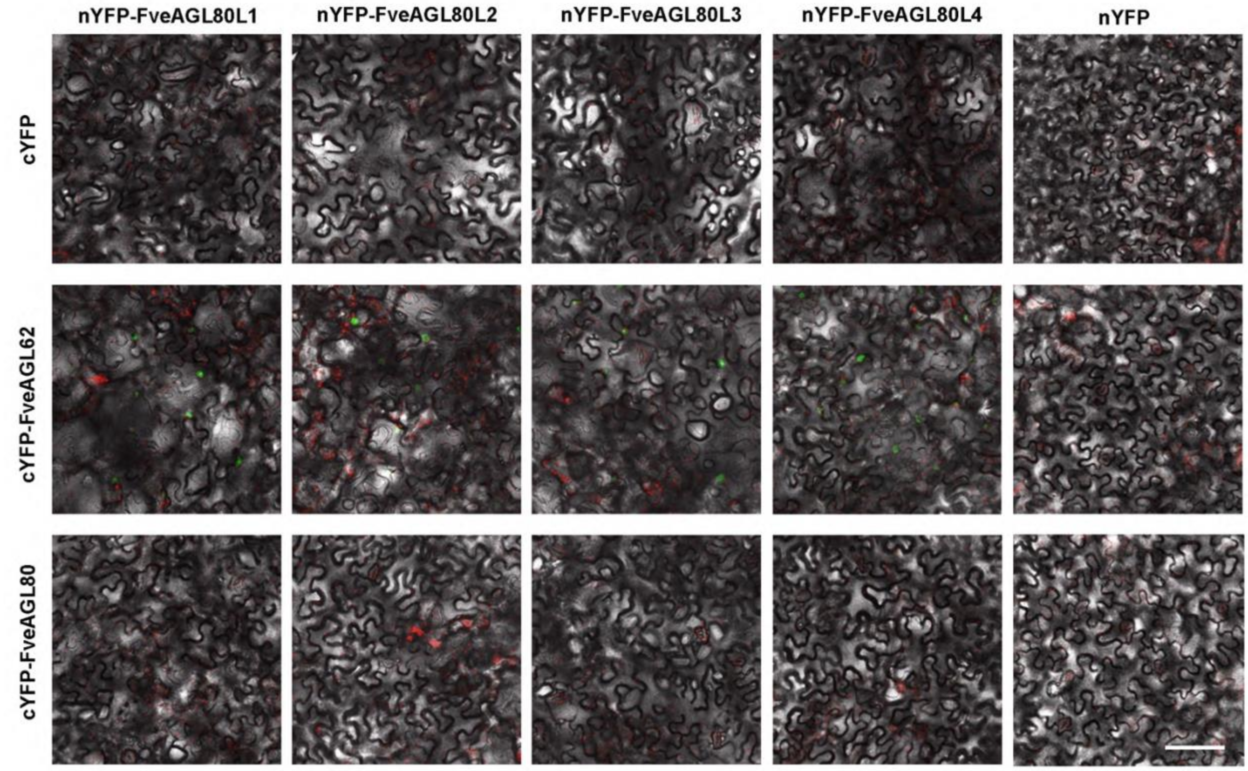

Supplementary Figure 4. BiFC assay showing the interaction between FveAGL62 and four FveAGL80_like proteins. Positive interactions between FveAGL62 and the four FveAGL80L proteins FveAGL80L1 (FvH4_6g21170), FveAGL80L2 (FvH4_7g09730), FveAGL80L3 (FvH4_6g08570), and FveAGL80L4 (FvH4_6g43410) are shown by the green fluorescent nuclei. No interaction is detected between FveAGL80 and any of the four FveAGL80L proteins. cYFP and nYFP vectors serve as negative controls. Scale bar, 100 μm.

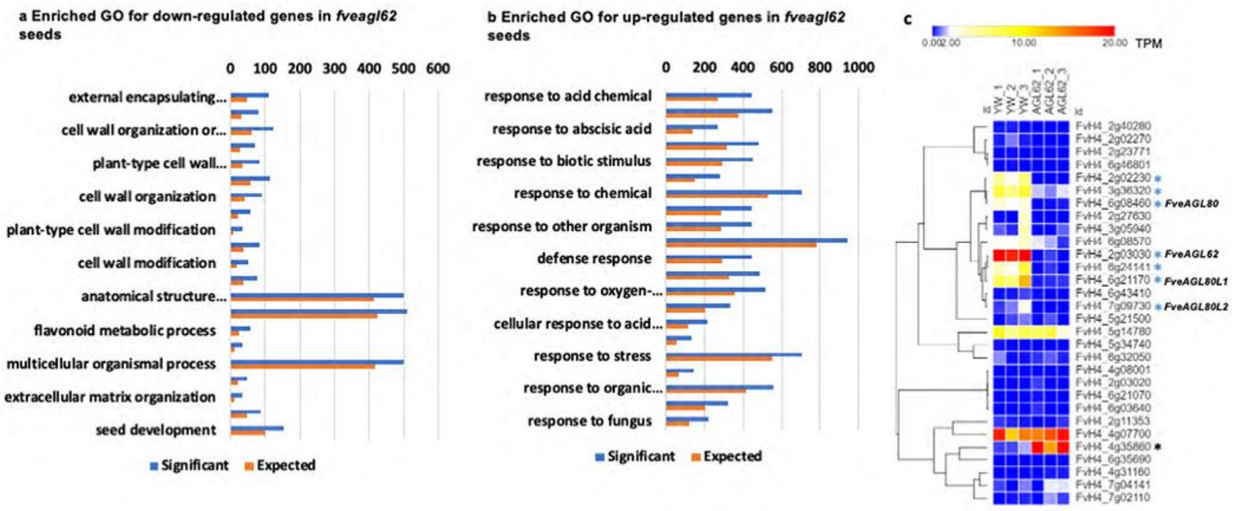

Supplementary Figure 5. Differential gene expression analysis between WT and *fveagl62* stage 2 seeds in *F. vesca.* a Top 21 enriched GO terms among 1102 genes that are down-regulated in *fveagl62* mutant seeds. **b** Top 21 Enriched GO terms among 1572 genes that are up-regulated in *fveagl62* mutant seeds. **c** Heatmap showing expression of all expressed type I MADS box genes in stage 2 seeds of WT (YW_1, YW_2, YW_3 replicates) and *fveagl62 (AGL62_1, AGL62_2, AGL62_3* replicates). Statistically significant DE genes are marked with * (blue * for down-regulated and black * for up-regulated genes in *fveagl62* mutant seeds).

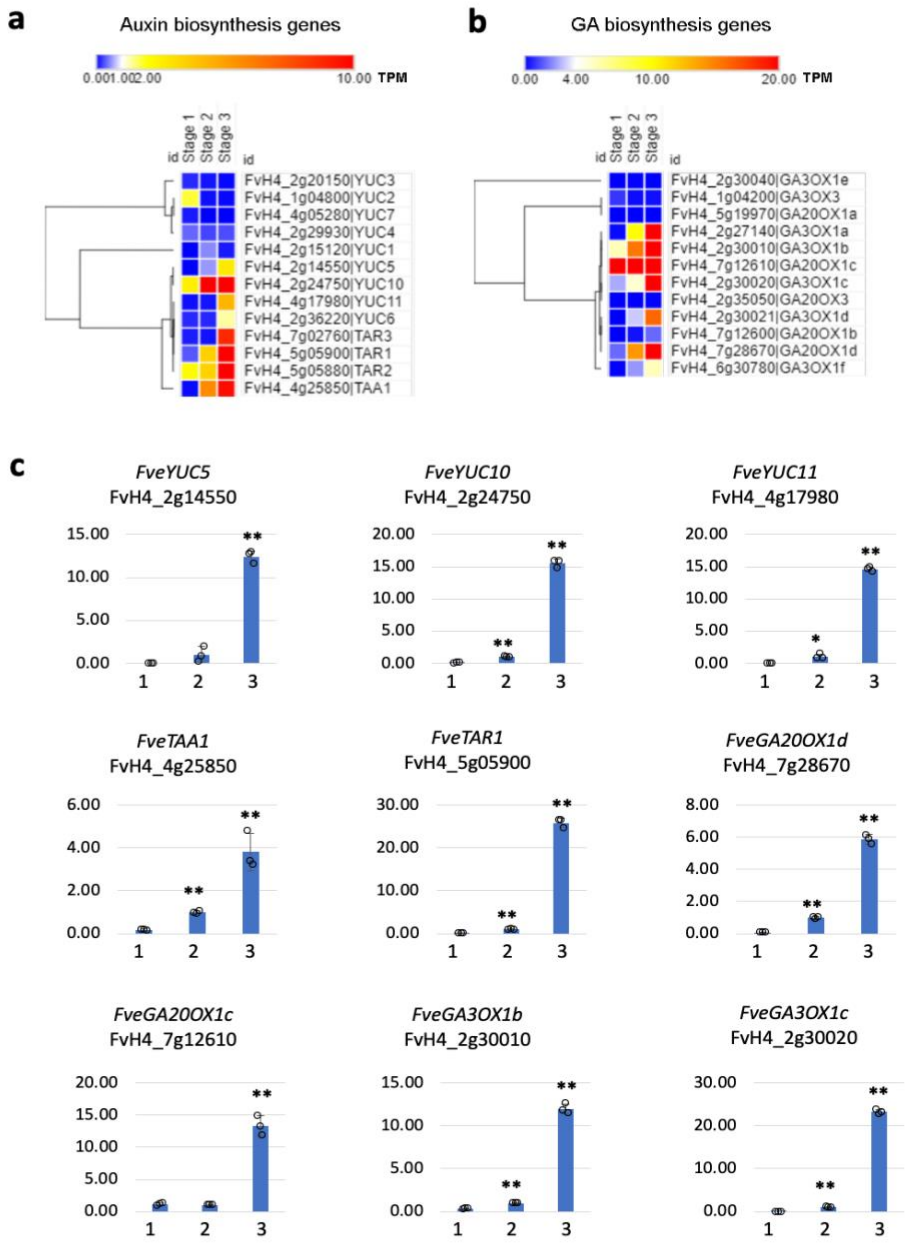

Supplementary Figure 6. *F. vesca* Auxin and GA biosynthesis gene expression in early-stage seeds. a Hierarchical clustering heatmap of auxin biosynthesis genes at three ovule/seed stages based on RNA-seq reads (TPM). Stage 1 is unfertilized ovule, stage 2 is fertilized seed, and stage 3 is ghost (endosperm and seedcoat). **b** Hierarchical clustering heatmap of GA biosynthesis genes at three ovule/seed stages based on RNA-seq reads (TPM). **c** RT-qPCR showing the relative transcript levels (Y-axis) of auxin and GA biosynthesis genes at the stages 1, 2, and 3. Significant difference from previous stage (two-tailed Student's *t*-test) is indicated by ** ($P < 0.01$) and * ($P < 0.05$). Error bars indicate standard deviation from three technical replicates. The experiment was repeated twice. The data shown in **a** and **b** are from re-analysis of a prior study[3].

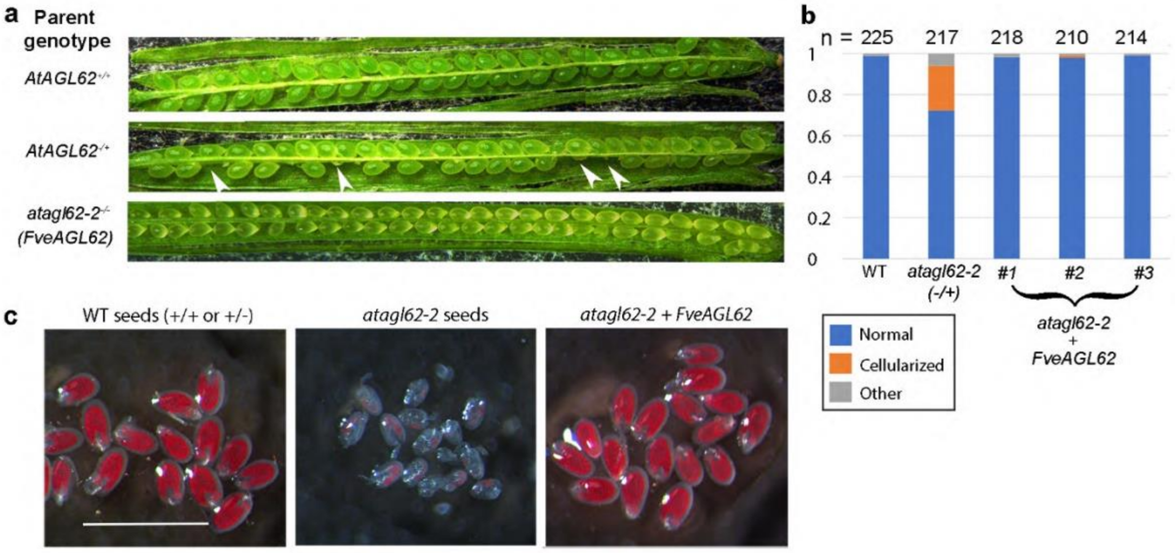

Supplementary Figure 7. Strawberry *FveAGL62* transgene rescues Arabidopsis *atagl62* mutant seeds. a Silique phenotype of the *atagl62-2* Arabidopsis mutants with or without the *FveAGL62* transgene. The silique (parent plant)'s genotype at the *AtAGL62* is shown to the left. A heterozygous *atagl62-2(-/+*) plant gives rise to 25% dead seeds shown as empty spaces (a few marked by arrowheads). Homozygous *atagl62-2* (-/-) plants are not viable unless they contain the *FveAGL62* transgene as shown. **b** Quantification of cellularized endosperms in the WT seeds, seeds derived from *atagl62-2(-/+)* parents, and seeds of three transgenic lines (#1, #2, and #3) of *atagl62-2* (-/-) containing the *FveAGL62* transgene. The number (n) of seeds examined is indicated above each bar. **c** Vanillin-stained Arabidopsis seeds of WT, *atagl62*-2 *(-/-)*, and *atagl62-2 (-/-)* containing the *FveAGL62* transgene driven by Arabidopsis *AtAGL62* cis-elements. Scale bar, 1 mm.

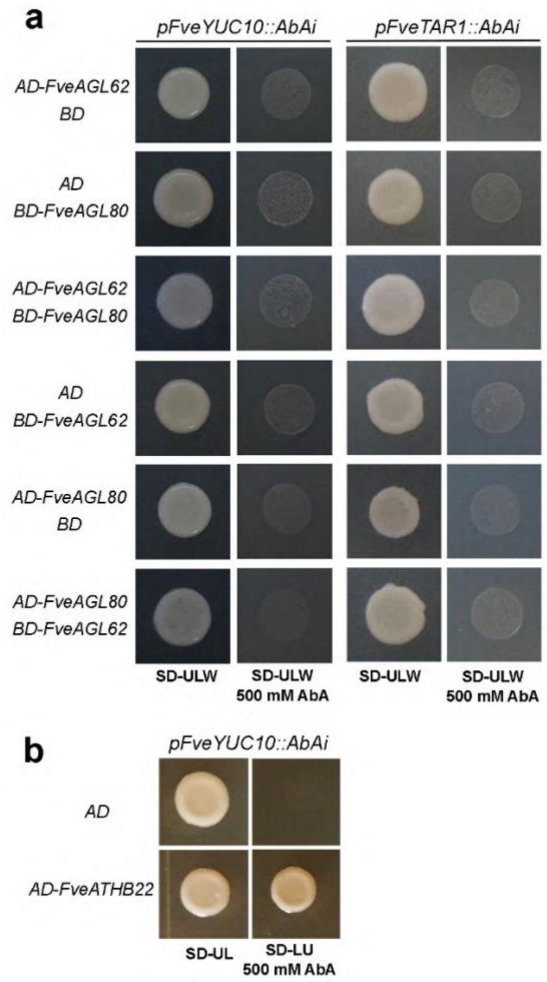

Supplementary Figure 8. FveAGL62 and FveAGL80 do not bind the promoters of auxin biosynthesis genes *FveYUC10* and *FveTAR1*. a Results of Y1H assays showing a lack of binding of FveAGL62 and FveAGL80, alone or in combination, to the promoters of *FveYUC10* and *FveTAR1*. **b** Confirmation of a Y1H screen result showing activation of the *pFveYUC10::AbAi* reporter by FveATHB22 (FvH4_1g20040).

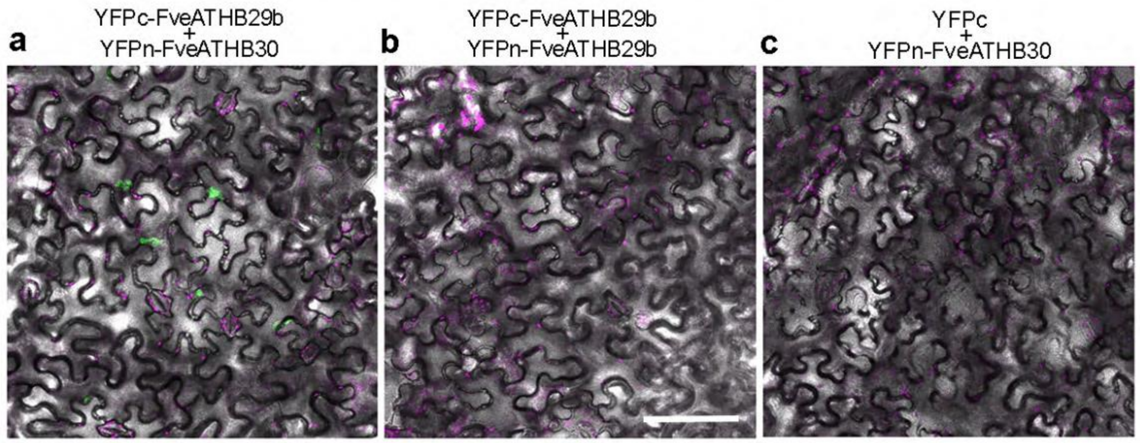

Supplementary Figure 9. BiFC data showing heterodimerization of FveATHB29b and FveATHB30. a Green fluorescent signal detected in the tobacco leaf infiltrated with agrobacteria containing *YFPc-FveATHB29b* and *YFPn-FveATHB30*. **b** a lack of green fluorescent signal when *YFPc-FveATHB29b* and *YFPn-FveATHB29b* were co-infiltrated, indicating a lack of homodimerization. **c** Negative control pairing the *YFPc* vector with *YFPn-FveATHB30*. Scale bar, 100 μm.

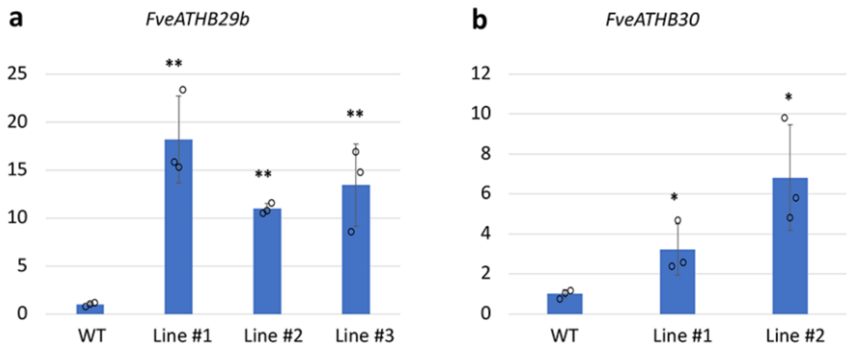

Supplementary Figure 10. *FveATHB29b-OE* and *FveATHB30-OE* transgene expression in *F. vesca*. a-b RT-qPCR showing relative transcript level (Y-axis) of *FveATHB29b* and *FveATHB30* genes in nontransgenic WT (YW5AF7) and respective *FveATHB29b-OE* (**a**) and *FveATHB30-OE* (**b**) transgenic lines. Significant difference from the WT (two-tailed Student's *t*-test) is indicated by ** ($P < 0.01$) in (**a**) or * ($P < 0.05$). Error bars indicate standard deviation of three technical replicates. The experiment was repeated twice with similar results.

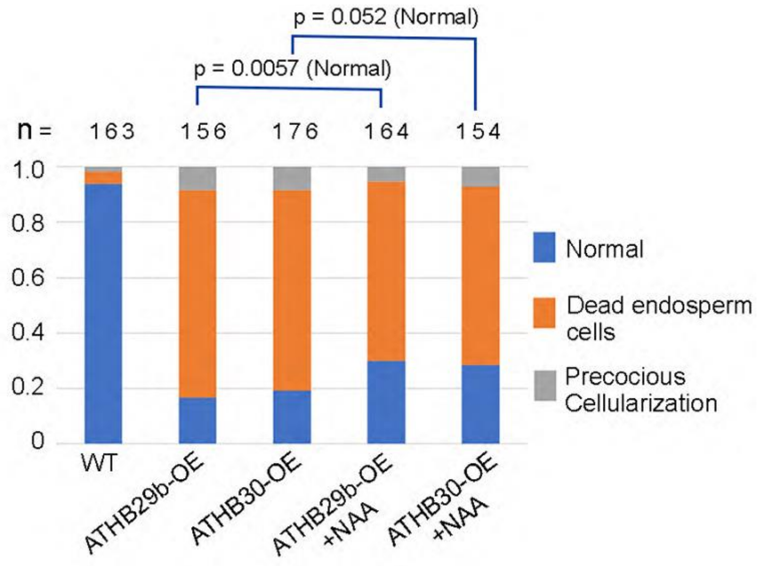

Supplementary Figure 11. Quantitation of endosperm defects in *FveATHB29b-OE* and *FveATHB30-OE* transgenic lines. Y-axis indicates percentage of seeds with the phenotype indicated by the color code. Stage 2 seeds of *FveATHB29b-OE* line #1 and *FveATHB30-OE* line #1 were analyzed under the confocal microscope to score endosperm phenotype. Number (n) of seeds analyzed per genotype is indicated above each bar. +NAA: auxin application. Significant difference in the "Normal" category (blue bar) between NAA-treated and nontreated seeds of the same transgenic line was evaluated with two-tailed Fisher's exact test with the p-value shown.

Reference

1. Shahan, R. *et al.* Consensus coexpression network analysis identifies key regulators of flower and fruit development in wild strawberry. *Plant physiology* **178**, 202–216 (2018).
2. Hawkins, C. *et al.* An eFP browser for visualizing strawberry fruit and flower transcriptomes. *Horticulture Research* **4**, 17029 (2017).
3. Kang, C. *et al.* Genome-Scale Transcriptomic Insights into Early-Stage Fruit Development in Woodland Strawberry Fragaria vesca. *The Plant Cell* **25**, 1960–1978 (2013).

**Supplementary Table 1: primers used in this study**		
**Primer name**	**Primer sequence 5'-3'**	**Experiment**
FveAGL62-gRNA1-F	ggcgagccagGTTTTAGAGCTAGAAATAGC	CRISPR/Cas9
FveAGL62-gRNA1-R	ttgcgcacccAATCACTACTTCGACTCTAG	CRISPR/Cas9
FveAGL62-gRNA3-F	CTAGAGTCGAAGTAGTGATTgCTATCACAGACTCCACGCAGGTTTTAGAGCTAGAAATAG	CRISPR/Cas9
FveAGL62-gRNA3-R	CTATTTCTAGCTCTAAAACCTGCGTGGAGTCTGTGATAGcGAgCTATACTGCAATGTTC	CRISPR/Cas9
FveAGL80-gRNA1-F	gctcGCGATGGACTCCCAACGGGGA	CRISPR/Cas9
FveAGL80-gRNA1-R	aaacTCCCCGTTGGGAGTCCATCGC	CRISPR/Cas9
MDC162-FveAGL62-GUS-F	GCCAAGCTCTAGTTAATTAAAAGACTGAACTCTTCAATTATC	GUS fusion reporter
MDC162-FveAGL62-GUS-R	CTCTAGAGTCGAGGCGCGCCAAGAAGAAGCGACCAAATGCTG	GUS fusion reporter
MDC162-FveAGL80-GUS-F	GCCAAGCTCTAGTTAATTAACTGAACTTGCTTTTGTACTAC	GUS fusion reporter
MDC162-FveAGL80-GUS-R	CTCTAGAGTCGAGGCGCGCCAAAGGAAAATTAAAGCCATTAG	GUS fusion reporter
GBKT7-EcoRI-FveAGL62-F	catatggccatggaggccgaattcATGGTGAGGAAAAGCAAGGGTC	Y2H
GBKT7-BamHI-FveAGL62-R	CCGCTGCAGGTCGACGGATCCTCAGAAGAAGCGACCAAATG	Y2H
GADT7-EcoRI-FveAGL80-F	gccatggaggccagtgaattcATGACTAGAAAGAAGGTGAAAC	Y2H
GADT7-BamHI-FveAGL80-R	CAGCTCGAGCTCGATGGATCCTCAAGGAAAATTAAAGCCATTAG	Y2H
PXY-FveAGL62-Gibson-F:	CGAGGACGCCGGCGGATCCATGGTGAGGAAAAGCAAGGGTC	BiFC
PXY-FveAGL62-Gibson-R:	GATACGAACGAAAGCTCTGCAGTCAGAAGAAGCGACCAAATGC	BiFC
PXY-FveAGL80-Gibson-F	CGAGGACGCCGGCGGATCCATGACTAGAAAGAAGGTGAAAC	BiFC
PXY-FveAGL80-Gibson-R	CTCTGCAGGTCGACTCTAGATCAAGGAAAATTAAAGCCATTAG	BiFC
AbAi-KpnI-FveYUC10p-F:	GAATTCGAGCTCGGTACCAAGAGAAGAAGGAGACAAACAC	Y1H
AbAi-SalI-FveYUC10p-R:	GCACATGCCTCGAGGTCGACTTTGCGGTGATGTATAGGAG	Y1H
AbAi-KpnI-FvTAR1pro-F	CTTGAATTCGAGCTCGGTACCGGCTAGCAAACATCAACATTG	Y1H
AbAi-SalI-FvTAR1pro-R	GCACATGCCTCGAGGTCGACTTTTGTAGAGCTATGGAAAG	Y1H
JH23-KpnI-FveAGL62-F	tatttacaattacaaggtaccATGGTGAGGAAAAGCAAG	Over expression
JH23-PacI-FveAGL62-R	TCATTAAAGCAGttaattaaTCAGAAGAAGCGACCAAATG	Over expression
JH23-KpnI-FveAGL80-F	tatttacaattacaaggtaccATGACTAGAAAGAAGGTG	Over expression
JH23-PacI-FveAGL80-R	TCATTAAAGCAGttaattaaTCAAGGAAAATTAAAGCC	Over expression
GADT7-EcoRI-FveATHB29b-F	ccatggaggccagtgaattcATGAACATATATTATAATGCCAAG	Y1H
GADT7-BamHI-FveATHB29b-R	cagctcgagctcgatggatccCTACTGGGTTGTGAGAGAAG	Y1H

GADT7-EcoRI-FveATHB30-F	ccatggaggccagtgaattcATGGATATAGCGCCTTCCATC	Y1H
GADT7-BamHI-FveATHB30-R	cagctcgagctcgatggatccTCAAGAAGAAGATGACGAAC	Y1H
JH23-KpnI-FveATHB30-F	tatttacaattacaaggtaccATGGATATAGCGCCTTCCATC	Over expression
JH23-PacI-FveATHB30-R	TCATTAAAGCAGttaattaaTCAAGAAGAAGATGACGAAC	Over expression
JH23-KpnI-FveATHB29b-F	tatttacaattacaaggtaccATGAACATATATTATAATGC	Over expression
JH23-PacI-FveATHB29b-R	TCATTAAAGCAGttaattaaCTACTGGGTTGTGAGAGAAG	Over expression
DLR-NotI-FveATHB29bpro-F	catgcgacgtcgGCGGCCGCCTACAACTAAATGAAGACG	LUC reporter
DLR-BamHI-FveATHB29bpro-R	CAAGAAAGCTGGGTCGGATCCATCTCTCTCTCTCTTTGGTTC	LUC reporter
DLR-NotI-FveATHB30pro-F	catgcgacgtcgGCGGCCGCATCCGTCAAAAGTACGCTAATG	LUC reporter
DLR-BamHI-FveATHB30pro-R	CAAGAAAGCTGGGTCGGATCCCACTCAAACATCTAACAG	LUC reporter
PXY106-BamHI-FveATHB30-F	CGAGGACGCCGGCGGATCCATGGATATAGCGCCTTCCATC	BiFC
PXY106-XbaI-FveATHB30-R	CTCTGCAGGTCGACTCTAGATCAAGAAGAAGATGACGAAC	BiFC
PXY105-BamHI-FveATHB29b-F	CTGTACAAGGCCGGCGGATCCATGAACATATATTATAATGC	BiFC
PXY105-XbaI-FveATHB29b-R	CTCTGCAGGTCGACTCTAGACTACTGGGTTGTGAGAGAAG	BiFC
PCR8-AtAGL62pro-Gibson-F	CAAAAAAGCAGGCTCCGAATTCttactgcaaaagtagtttgtctc	Ectopic expression
AtAGL62pro-FveAGL62-Gibson-R	CCTTGCTTTTCCTCACCATaagcttttttagtgatatttgagaagcta	Ectopic expression
AtAGL62pro-FveAGL62-Gibson-F	tagcttctcaaatatcactaaaaaagcttATGGTGAGGAAAAGCAAGG	Ectopic expression
PCR8-FveAGL62-Gibson-R	gttagTTAATTAACggcgcgccTCAGAAGAAGCGACCAAATG	Ectopic expression
FveAGL62-AtAGL62ter-F	CATTTGGTCGCTTCTTCTGAggcgcgcccccctttgttgtgtgatccaac	Ectopic expression
PCR8-AtAGL62ter-R	CAAGAAAGCTGGGTCGGATCCGCGGCCGCCAATCACTAGGGTTTGA tac	Ectopic expression
AtAGL62-Salk-FP	tgcataccatatattgattg	T-DNA genotype
AtAGL62-Salk-RP	TGTTTCCAAAGGGTGGTAATG	T-DNA genotype
LBb1.3	ATTTTGCCGATTTCGGAAC	T-DNA genotype
FvePP2a-qPCR-F	ATGTCTGTGGTTGATGAACCG	RT-qPCR
FvePP2a-qPCR-R	CCAATCCTACATAATGACTCCAC	RT-qPCR
FveTAR1-qPCR-F	CACAAATTTGCTTATTCTGTC	RT-qPCR
FveTAR1-qPCR-R	TTGTTGGGTC ACCATGGTTG	RT-qPCR
FveYUC10-qPCR-F	CAAGGACTTCAATCATTGTTC	RT-qPCR
FveYUC10-qPCR-R	GATACTTACCGTACTTGATC	RT-qPCR
FveTAA1-qPCR-F	GGCACAAGAAGGTCTAGCGT	RT-qPCR
FveTAA1-qPCR-R	TTGTGCACTTGTCCCCCATC	RT-qPCR

FveATHB29b-qPCR-FP	AGATGAGATTGAGAGGTTCTG	RT-qPCR
FveATHB29b-qPCR-RP	TTGTGAGAGAAGAGGCATTG	RT-qPCR
FveATHB30-qPCR-FP	ATGGATATAGCGCCTTCCATC	RT-qPCR
FveATHB30-qPCR-RP	TCACGGGTATGTTACTGTTG	RT-qPCR
FvYUC11-qPCR-F	GAAAGATTAAGGTTTTCTCCG	RT-qPCR
FvYUC11-qPCR-R	GACCTTATAATCCTTGAGCC	RT-qPCR
FvYUC5-qPCR-F	TCGAGCAAGTATGACAATGG	RT-qPCR
FvYUC5-qPCR-R	AGAACATGTGTCAGGCTACG	RT-qPCR
FveGA3OX1b (2g30010)-qPCR-F	CCA GCT GAA CAA AAG TTG AA	RT-qPCR
FveGA3OX1b (2g30010)-qPCR-R	TGC TTG CTT CTG GTA ATC AT	RT-qPCR
FveGA3OX1c (2g30020)-qPCR-F	CAA TTG ATC ATG CTA AGG CG	RT-qPCR
FveGA3OX1c (2g30020)-qPCR-R	AGA GTT CAA CTG TAA AGC CA	RT-qPCR
FvGA20OX1d (7g28670)-qPCR-F	GATGAATTCGGGAGGGTGTAC	RT-qPCR
FvGA20OX1d (7g28670)-qPCR-R	GTAATTCAACCTCATTATCG	RT-qPCR
FvGA20OX1c (7g12610)-qPCR-F	CAGACTTAACACTGGGAACTG	RT-qPCR
FvGA20OX1c (7g12610)-qPCR-R	CACAGAGCCATAAAAGTATC	RT-qPCR
PXY106-BamHI-FvAGL80L1-F	CGAGGACGCCGGCGGATCCATGGCAAGAAAGAAGGTGAAATTG	BiFC
PXY-XbaI-FvAGL80L1-R	CTCTGCAGGTCGACTCTAGACTAGATCGTCAGCCTTTTAATAAC	BiFC
PXY106-BamHI-FvAGL80L2-F	CGAGGACGCCGGCGGATCCATGGCTAGAAAGAAAGTGAG	BiFC
PXY-XbaI-FvAGL80L2-R	CTCTGCAGGTCGACTCTAGATTATGCAGTTTGGTTCTCGTTATC	BiFC
PXY106-BamHI-FvAGL80L34-F	CGAGGACGCCGGCGGATCCATGACTAGAAAGAAGGTGAAAC	BiFC
PXY-XbaI-FvAGL80L3-R	CTCTGCAGGTCGACTCTAGACTAAGGAAAATTAAAGCCGTC	BiFC
PXY-XbaI-FvAGL80L4-R	CTCTGCAGGTCGACTCTAGATCAAGGCAAATTATTGCCACC	BiFC
AbAi-KpnI-GENE03815pro-1200bpF	CTTGAATTCGAGCTCGGTACCCTTTACTGGCGAGAGCAC	Y1H
AbAi-SalI-GENE03815pro-581bp-R	GCACATGCCTCGAGGTCGACTCGGTGTATTTTTCCCTGAC	Y1H
ABAI-GENE09326pro-3-F	CTTGAATTCGAGCTCGGTACCCCAATAACAATACGTTTG	Y1H
AbAi-SalI-GENE09326pro-R	GCACATGCCTCGAGGTCGACATCTCTCTCTCTCTTTGGTTC	Y1H
