# Supplementary material for: Mechanism of fertilization-induced auxin synthesis in the endosperm for seed and fruit development
Source: Nat Commun. 2022 Jul 9;13:3985. doi: 10.1038/s41467-022-31656-y (PMC9271072; doi:10.1038/s41467-022-31656-y)
Supplement: Supplementary file 3 — Description of Additional Supplementary Files [file 41467_2022_31656_MOESM3_ESM.pdf]

Description of Additional Supplementary Files

File Name: Supplementary Movie 1
Description: Confocal z-stack of an F. vesca wild type stage 2 seed.

File Name: Supplementary Movie 2
Description: Confocal z-stack of an F. vesca fveagl62 stage 2 seed.

File Name: Supplementary Movie 3
Description: Confocal z-stack of an F. vesca fveagl62 stage 2 seed mock-treated.

File Name: Supplementary Movie 4
Description: Confocal z-stack of an F. vesca fveagl62 stage 2 seed treated with NAA.

File Name: Supplementary Data 1
Description: Gene list in the cluster#76 of consensus co-expression network 90.

File Name: Supplementary Data 2
Description: Differentially expressed gene list between WT (YW) and fveagl62 seeds.

File Name: Supplementary Data 3
Description: Enriched GO terms for up- and down-regulated genes in fveagl62 seeds.
